# Sphingosine Phosphate Lyase Regulates Murine Embryonic Stem Cell Proliferation and Pluripotency through an S1P_2_/STAT3 Signaling Pathway

**DOI:** 10.3390/biom3030351

**Published:** 2013-06-24

**Authors:** Gaelen S. Smith, Ashok Kumar, Julie D. Saba

**Affiliations:** Children’s Hospital Oakland Research Institute, 5700 Martin Luther King Jr. Way, Oakland, CA 94609, USA; E-Mails: sciencism@gmail.com (G.S.S.); ashok.biochemistry@aiimsbhopal.edu.in (A.K.)

**Keywords:** sphingosine-1-phosphate, sphingosine phosphate lyase, embryonic stem cell, S1P_2_, pluripotency, proliferation, STAT3

## Abstract

Sphingosine-1-phosphate (S1P) is a bioactive sphingolipid that activates a family of G protein coupled-receptors (GPCRs) implicated in mammalian development, angiogenesis, immunity and tissue regeneration. S1P functions as a trophic factor for many cell types, including embryonic stem cells (ESCs). Sphingosine phosphate lyase (SPL) is an intracellular enzyme that catalyzes the irreversible degradation of S1P. We found SPL to be highly expressed in murine ESCs (mESCs). To investigate the role of SPL in mESC biology, we silenced SPL in mESCs via stable transfection with a lentiviral SPL-specific short hairpin RNA (shRNA) construct. SPL-knockdown (SPL-KD) mESCs showed a 5-fold increase in cellular S1P levels, increased proliferation rates and high expression of cell surface pluripotency markers SSEA1 and OCT4 compared to vector control cells. Compared to control mESCs, SPL-KD cells showed robust activation of STAT3 and a 10-fold increase in S1P_2_ expression. Inhibition of S1P_2_ or STAT3 reversed the proliferation and pluripotency phenotypes of SPL-KD mESCs. Further, inhibition of S1P_2_ attenuated, in a dose-dependent fashion, the high levels of OCT4 and STAT3 activation observed in SPL-KD mESCs. Finally, we showed that SPL-KD cells are capable of generating embryoid bodies from which muscle stem cells, called satellite cells, can be isolated. These findings demonstrate an important role for SPL in ESC homeostasis and suggest that SPL inhibition could facilitate *ex vivo* ESC expansion for therapeutic purposes.

## 1. Introduction

Embryonic stem cells (ESCs) are pluripotent cells derived from the inner cell mass of preimplantation embryos [1]. Stem cells are unique in their ability to both self-renew and undergo differentiation, giving rise to the cells that comprise the three germ layers of a developing embryo: mesoderm, ectoderm, and endoderm. There is great interest in using ESCs and other pluripotent stem cells for tissue regeneration in human disease. As such, elucidating the mechanisms by which ESCs achieve self-renewal is an important goal.

ESCs maintain pluripotency through signaling networks that are activated downstream of leukemia inhibitory factor receptor (LIFR) [2]. Upon association with the cytokine leukemia inhibitory factor (LIF), the LIFR heterodimerizes with the glycoprotein 130 (gp130) trans-membrane receptor. Downstream pathways of gp130-dependent signaling networks include janus kinase (JAK)-signal transducer and activator of transcription (STAT), mitogen-activated protein kinase (MAPK) and phosphatidylinositide 3-kinase (PI_3_K), each of which differentially modulates the expression of the three major regulatory transcription factors of ESCs: Octamer-binding protein 4 (OCT4), NANOG, and SRY (sex determining region Y)-box 2 (SOX2). These transcription factors are critical to controlling ESC self-renewal and maintenance of pluripotency through the regulation of downstream target genes [3]. 

Sphingosine-1-phosphate (S1P) is a bioactive sphingolipid involved in cell survival, proliferation, migration and inflammatory signaling in many cell types [4]. S1P plays critical roles in a variety of cellular processes, including DNA synthesis [5], inhibition of apoptosis [6], thymic egress of T-cells [7], angiogenesis [8], muscle regeneration [9], and stem cell trafficking [10]. In addition to these functions, S1P has been shown to function as a trophic factor in ESCs, promoting their survival as well as the maintenance of ESC pluripotency [11]. 

S1P is produced by the phosphorylation of sphingosine through the actions of sphingosine kinase enzymes SphK1 and SphK2 [12]. S1P can be dephosphorylated by S1P phosphohydrolases, or irreversibly catabolized into ethanolamine phosphate and hexadecenal by sphingosine phosphate lyase (SPL), a ubiquitously expressed pyridoxal 5’-phosphate-dependent enzyme [13]. S1P binds extracellularly to a family of five G-coupled receptors, S1P_1-5_. S1P receptor expression varies among tissues. S1P_1-3_ receptors are found in all tissues of the body. S1P_4_ and S1P_5_ receptors are tissue-specific, and their expression is restricted to cells comprising brain and nervous tissue, as well as hematopoietic precursors and ESCs [11,14]. Human ESCs identified by expression of extracellular stem cell marker Tra-1-60 were shown to express S1P_1-3_ [15], whereas mouse ESCs were found to express S1P_1-3_ and S1P_5_ but not S1P_4_ [14].

S1P has a pro-proliferative effect in both human ESCs (hESCs) [16] and mouse ESCs (mESCs) [14], as well as in other multipotent stem cells, including neural progenitor cells [17,18] and bone marrow-derived stem cells [19,20]. In mESCs, the addition of S1P and platelet derived growth factor (PDGF) in serum-free conditions allows maintenance of an undifferentiated state, and MAPKK inhibition with a small molecule inhibitor U0126 reverses this effect, indicating that S1P-MAPK signaling regulates mESC pluripotency [16]. However, inhibition of S1P_1-3_ receptors does not affect MAPK phosphorylation [14]. Thus the S1P-MAPK effect is likely not mediated through S1P_1-3_. In hESCs, incubation with S1P leads to decreased apoptosis, increased DNA synthesis and increased cell proliferation [15,21]. Incubation of hESCs with S1P or PDGF also leads to increased ERK1/2 and/or AKT signaling [21,22]. Surprisingly, in mESCs, suppression of ERK signaling leads to increased proliferation [23]. This suggests that the signaling pathways downstream of S1P and PDGF, through ERK and AKT, are independent of one another in hESCs and in mESCs. In mESCs, the extent of S1P signaling through pathways other than MAPK and PI_3_K has yet to be defined.

STAT3 is part of the STAT family of transcription factors, which is comprised of STAT1-6. These proteins are activated upon phosphorylation of STAT Src-homology 2 (SH2) domains by Janus Kinases (JAKs) [24]. In ESCs, STAT3 signaling is initiated through binding of LIF to its receptor and resulting activation of JAK1/2 [25]. JAKs phosphorylate four distinct gp130 tyrosine residues, triggering the binding and phosphorylation of STAT3 at its SH2 domain to the JAK/gp130 complex. Upon phosphorylation of the Tyrosine 705 residue of STAT3 by JAK, STAT proteins dimerize and translocate to the nucleus, binding promoter regions and regulating DNA transcription [26]. STAT3-induced dysregulation of S1P_1_ signaling has been implicated in tumor growth [27]. STAT signaling is critical to the regulation of ESC pluripotency [28]. In mESCs, STAT3 functions as a transcription factor with over 900 putative binding sites [29]. ES cells which express inhibitory STAT mutants down-regulate pluripotency transcription factors and are more prone to differentiation than null mutants [25]. In ESCs, inhibition of STAT3 signaling promotes differentiation and blocks self-renewal [28].

In this study, we explored the role of SPL in ESC biology by generating a pair of mESC lines, one in which SPL was silenced by stable transduction with a lentiviral construct expressing mSPL-specific shRNA and the other transduced with empty lentiviral vector. We observed that SPL knockdown (SPL-KD) cells exhibited increased proliferation and expression of the pluripotency markers SSEA1 and OCT4 compared to control cells. The phenotype of SPL-KD cells was associated with increased levels of STAT3 activation and increased gene expression of S1P_2_ compared to control cells. Further, chemical inhibition of either STAT3 or S1P_2_ attenuated the high SSEA1 expression and growth advantage of SPL-KD cells to control cell levels. Finally, inhibition of S1P_2_ reduced STAT3 activation in SPL-KD cells in a dose-dependent manner commensurate with SSEA1 downregulation. Our results suggest that SPL modulates mESC proliferation and pluripotency through an S1P_2_-STAT3-dependent signaling pathway. 

## 2. Results and Discussion

### 2.1. Silencing of SPL in mESCs

**Figure 1:**
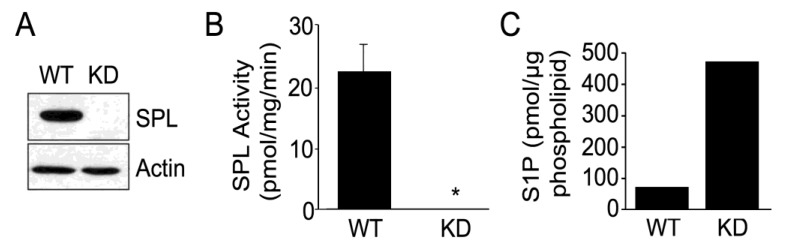
Generation of sphingosine phosphate lyase (SPL) murine embryonic stem cells (mESCs) knockdown and vector control cell lines. (**A**) Wild type (WT) E14 mES cells containing empty pLKO.1 vector show robust SPL expression by immunoblotting. SPL was undetectable by immunoblotting in mESCs in which SPL was knocked down (KD) using *Sgpl1* short hairpin RNA (shRNA) expressing construct in lentiviral vector pLKO.1. These results represent three separate experiments; (**B**) SPL enzyme activity is undetectable in whole cell extracts from SPL-KD mESCs. * For WT *vs.* KD, *p <* 0.05; (**C**) S1P levels quantified by mass spectrometry in WT and SPL-KD mESCs.

We have shown that mouse embryos exhibit robust expression of SPL in many developing tissues [30]. In order to investigate the function of SPL in mESC biology during the earliest stages of mammalian development, we established an E14 mESC cell line in which SPL expression was silenced. This was accomplished through stable transduction of mESCs with a lentiviral vector that drives expression of an shRNA directed against mSPL, generating clonal SPL-KD mESC lines. Wild type (WT) control E14 cell lines harboring empty vector cassettes were also generated. To confirm the silencing of SPL in SPL-KD mESCs, western blotting of whole cell extracts was performed using a rabbit polyclonal antibody against SPL. WT mESCs propagated under routine proliferation conditions exhibited appreciable amounts of SPL protein. However, established SPL-KD mESC clones did not have detectable expression of SPL, as shown by the results from a representative clone of each type in Figure 1A. To further confirm the SPL silencing, we measured the SPL enzyme activity in WT and SPL-KD mESCs using a ω-labeled BODIPY–S1P substrate. As shown in Figure 1B, compared to moderate SPL activity detected in WT mESCs, SPL silencing resulted in a lack of detectible SPL activity. To investigate the impact of SPL silencing on S1P intracellular mESC S1P levels, S1P levels were measured in whole cell extracts of WT and SPL-KD cells using mass spectrometry. As shown in Figure 1C, S1P levels in SPL-KD mESCs were higher than in WT mESCs.

### 2.2. SPL Silencing Enhances mESC Proliferation and Pluripotency

**Figure 2:**
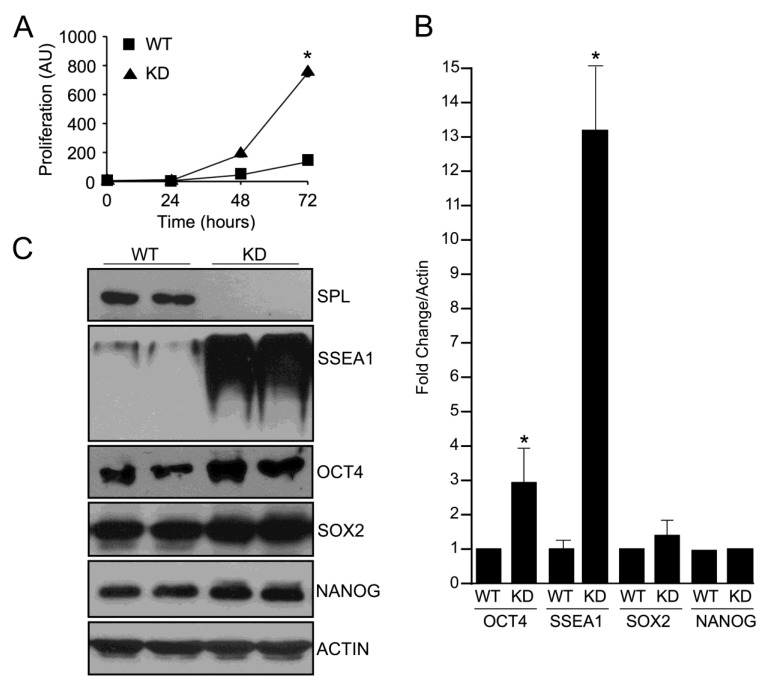
Effects of SPL silencing on mESC proliferation and pluripotency marker expression. (**A**) Proliferation was determined by serial cell counts of exponentially growing cultures of SPL-KD (closed triangle) and vector control (closed square) mESCs. * For WT *vs.* KD at 72 h, *p <* 0.05; (**B**) Quantification of pluripotency markers SSEA1, OCT4, SOX2 and NANOG protein expression relative to Actin loading control as determined by ImageJ software analysis. * For WT *vs.* KD expression of OCT4 and SSEA1, *p <* 0.05; (**C**) Protein levels of pluripotency markers SSEA1, OCT4, SOX2 and NANOG and Actin control were measured by immunoblotting whole cell extracts of SPL-KD and vector control mESCs. Shown is representative immunoblot used for quantification of results depicted in (**B**). These results are representative of at least three separate experiments.

**Figure S1:**
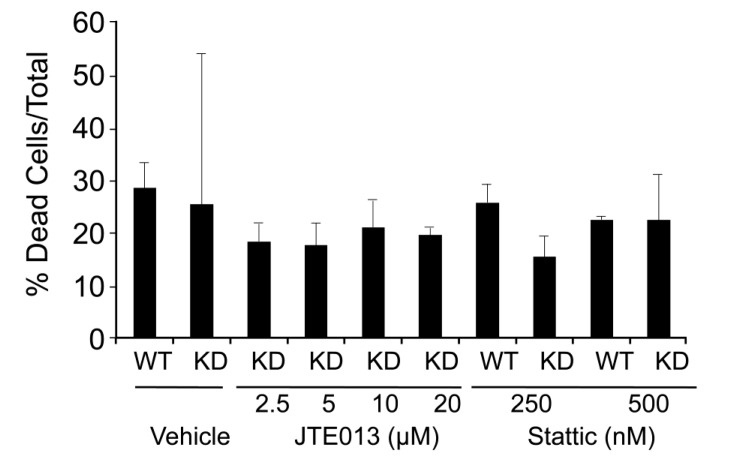
S1P_2_ antagonist JTE013 and STAT3 inhibitor Stattic do not induce necrotic cell death. Trypan blue staining was performed on cell cultures of SPL-KD and vector control mESCs grown in the presence of S1P_2_ antagonist JTE013 and STAT3 inhibitor Stattic under similar conditions as described for proliferation assays shown in Figure 2, Figure 3 and Figure 4. Minimal Trypan Blue Dye uptake was observed.

To assess whether SPL silencing affected cell growth, proliferation rates of WT and SPL-KD lines were measured at a variety of seeding densities. SPL-KD cells exhibited an increased proliferation rate in comparison to WT (Figure 2A), with no significant difference in cell death as determined by Trypan Blue Dye staining (Supplemental Figure S1). Examination of cell morphology did not reveal evidence of increased differentiation within SPL-KD mESC colonies (data not shown). In order to assess the pluripotency of each cell line, western blotting was performed with antibodies against stage-specific embryonic antigen-1 (SSEA1), a plasma membrane marker of mESC pluripotency, as well as for OCT4, SOX2 and NANOG. SPL-KD cells exhibited significantly increased expression levels of both SSEA1 and OCT4, with the greatest effect on SSEA1, as shown by western blot autoradiogram and quantified by ImageJ software analysis (Figure 2B,C). No consistent difference was observed in expression levels of SOX2 and NANOG between the two cell lines. Increased expression of OCT4 was present in multiple SPL-KD clones (Figure 2B,C), indicating that this was not an artifact of gene perturbation during lentiviral integration of mESCs.

**Figure 3:**
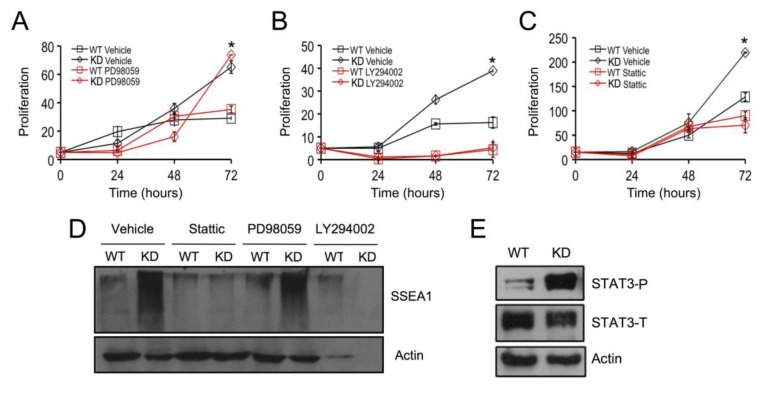
SPL silencing promotes proliferation and pluripotency marker expression through STAT3 activation in mESCs. (**A**) SPL-KD and WT mESCs were grown to confluence, trypsinized, counted, and seeded at 75,000 cells/mL. Exponentially growing cultures of SPL-KD and WT mESCs were then treated with 10 µM PD98059 for 72 h. Cell proliferation was determined at the indicated time points by cell counting of vehicle-treated SPL-KD (black open diamond), vehicle-treated WT cells (black open square), inhibitor-treated SPL-KD (red open diamond), and inhibitor-treated WT (red open square).* For inhibitor treated cells, difference between SPL-KD and WT cells remains significant, *p <* 0.05; (**B**) SPL-KD and WT mESC cultures were prepared as in (**A**) but were treated instead with 1 µM LY294002 for 72 h, followed by cell counting at indicated time points. * For inhibitor treated cells, difference between SPL-KD and WT cells is no longer significant; (**C**) SPL-KD and WT mESC cultures were prepared as in (**A**) but were treated instead with 500 nM Stattic for 72 h, followed by cell counting at indicated time points. * For inhibitor treated cells, difference between SPL-KD and WT cells is no longer significant; (**D**) SPL-KD and WT mESC cultures were prepared as in (**A–C**), and cells were harvested at 72 h. Whole cell extracts were prepared and evaluated by immunoblotting for the proliferation marker SSEA1 with actin serving as a loading control. Note that LY284002 treatment resulted in cell death of both SPL-KD and vector control cells, shown by absence of actin; (**E**) Phosphorylated (activated) STAT3 (STAT3-P) and total STAT3 (STAT3-T) levels were measured by immunoblotting whole cell extracts of SPL-KD and WT mESCs. Actin was used as a loading control. These results are representative of at least three experiments.

### 2.3. SPL Silencing Acts via STAT3 Signaling to Enhance mESC Proliferation and Pluripotency

To identify the critical downstream target(s) responsible for the effects of SPL silencing in mESCs, proliferation assays were carried out in the presence or absence of small molecule inhibitors of MEK1 (PD98059), PI_3_K (LY294002), and STAT3 (Stattic) signaling. Following inhibition of MAPK signaling by incubation for 72 h with 10 µM PD98059, both SPL-KD and WT cell types exhibited slightly increased rates of proliferation in comparison to controls (Figure 3A), in agreement with previous studies [23]. Inhibition of PI_3_K signaling by incubation for 72 h with 1 µM LY294002 completely ablated growth in both WT and SPL-KD mESCs (Figure 3B), implicating this signaling pathway as critical to mESC survival. Interestingly, in the presence of 500 nM of the STAT3 inhibitor Stattic for 72 h, SPL-KD mESCs exhibited a markedly decreased proliferation rate compared to vehicle-treated SPL-KD cells (Figure 3C). In contrast, WT mESCs showed only modest reduction in growth in response to STAT3 inhibition. Use of the STAT3 inhibitor at two different concentrations did not cause cell death as determined by Trypan Blue Dye staining, ruling out the possibility that inhibited mESC growth following STAT3 inhibition was due to non-specific cytotoxicity (Supplemental Figure S1). 

To explore the role of MAPK, PI_3_K and STAT3 signaling in mediating the increased pluripotency observed in SPL-KD cells, WT and SPL-KD cells were treated with PD98059, LY294002 and Stattic as described above, whole cell extracts were harvested after 72 h incubation, and western blotting was performed to measure SSEA1 expression as an indicator of pluripotency. As shown in Figure 3D, the high levels of SSEA1 expression observed in SPL-KD cells were attenuated when STAT3 activity was inhibited, whereas inhibition of MAPK had no effect, and PI_3_K inhibition resulted in complete cell death at 72 h. These results implicated STAT3 signaling in mediating the SPL-KD phenotypes of high pluripotency marker expression and increased growth advantage. Therefore, it became important to determine whether SPL silencing influences STAT3 signaling. Toward that end, western blotting using an antibody that recognizes the activated form of STAT3, phosphorylated on Tyr 705 (STAT3-P) was performed on whole cell extracts of WT and SPL-KD cells. Importantly, the levels of STAT3-P were elevated in SPL-KD mESCs compared to control mESCs, whereas total STAT3 levels (STAT3-T) were unaffected by SPL status (Figure 3E). 

### 2.4. SPL Silencing Acts via S1P_2_ to Enhance mESC Proliferation and Pluripotency

**Figure 4:**
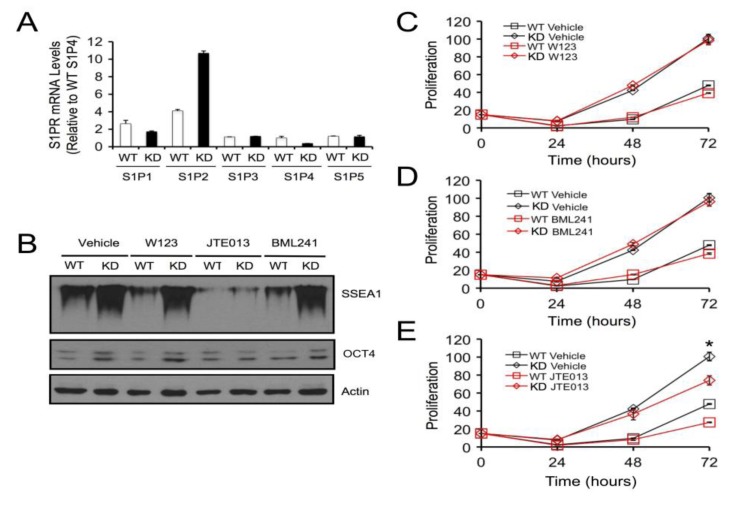
S1P_2_ is required to promote proliferation and pluripotency marker expression mediated by SPL silencing in mESCs. (**A**) Expression levels of S1P_1–5_ were determined by qRT-PCR in total RNA isolated from SPL-KD and WT mESCs grown under proliferation conditions; (**B**) SPL-KD and WT mESCs were grown to confluence, trypsinized, counted, and seeded at 75,000 cells/mL. Cells were grown in the presence of 20 µM of the S1P_1_ antagonist W123, 5 µM of the S1P_2_ antagonist JTE013, or 10 µM of the S1P_3_ antagonist BML241 (Cayman Chemical). Fresh media and inhibitors were added at 24-h time points. Cells were harvested at 72 h, and whole cell extracts were prepared and used for immunoblotting to measure proliferation antigen SSEA1, OCT4 and actin loading control; (**C**) SPL-KD and WT mESC cell cultures were prepared as in (**B**), treated with 20 µM W123. Cell proliferation was determined at the indicated time points by cell counting of vehicle-treated SPL-KD (black open diamond), vehicle-treated WT cells (black open square), antagonist-treated SPL-KD (red open diamond), and antagonist-treated WT (red open square). S1P_1_ antagonist W123; (**D**) SPL-KD and WT mESCs were prepared as in (**A**) but were treated instead with S1P_3_ antagonist BML241; (**E**) SPL-KD and WT mESCs were prepared as in (**A**) but were treated instead with S1P_2_ antagonist JTE013. These results are representative of three separate experiments. * For 72 h, *p <* 0.05 for SPL-KD cells with vehicle *vs.* JTE013.

In order to determine whether SPL’s role in mESC biology is mediated through S1P/S1P receptor signaling, WT and SPL-KD mESCs were first evaluated for S1P receptor expression levels by qRT-PCR. As shown in Figure 4A, all five S1P receptors were expressed in both WT and SPL-KD mESCs. Further, in both E14 mESC lines S1P_2_ was the most highly expressed receptor subtype, followed by S1P_1_, with low levels of S1P_3-5_ observed in both cell types. Interestingly, however, SPL-KD cells showed a significant shift in the representation of receptor subtypes, with lower S1P_1_ and higher S1P_2_ expression compared to WT mESCs. To evaluate the role of S1P receptors in mediating the impact of SPL silencing on pluripotency, WT and SPL-KD mESCs were grown in the presence of specific antagonists of S1P_1_ (20 µM W123), S1P_2_ (5 µM JTE013), and S1P_3_ (10 µM BML241) or vehicle control. Whole cell extracts were harvested at 72 h and evaluated for expression of the pluripotency markers SSEA1 and OCT4. As shown in Figure 4B, inhibition of S1P_1_ and S1P_3_ receptor signaling with W123 and BML241 had little or no impact on the high levels of SSEA1 and OCT4 expression observed in SPL-KD mESCs. However, SSEA1 expression of both SPL-KD and WT mESCs were decreased following incubation with S1P_2_ receptor antagonist JTE013 (Figure 4B). Similarly, treatment of SPL-KD cells with the S1P_2_ receptor inhibitor JTE013 abolished the difference in OCT4 expression between the two cell lines (Figure 4B). To determine whether S1P receptor signaling was also responsible for the impact of SPL silencing on mESC proliferation, proliferation assays were carried out in the presence or absence of antagonists of S1P_1_, S1P_2_, and S1P_3_ signaling. Following inhibition of S1P_1_ and S1P_3_ signaling by incubation for 72 h with W123 or BML241, SPL-KD and WT mESCs proliferated at rates similar to their corresponding vehicle-treated controls (Figure 4C,D). In contrast, WT and SPL-KD cells treated with JTE013 to inhibit S1P_2_ signaling exhibited a decrease in proliferation rate compared to the corresponding vehicle-treated mESCs, with SPL-KD cells exhibiting a significant reduction in growth after JTE013 treatment (Figure 4E). Treatment of mESCs with four different concentrations of JTE013 did not cause cell death as determined by Trypan Blue Dye staining, ruling out the possibility that reduced mESC growth following S1P_2_ antagonism was due to non-specific cytotoxicity (Supplemental Figure S1). 

### 2.5. S1P_2_ Activates STAT3 Signaling in mESCs

**Figure 5:**
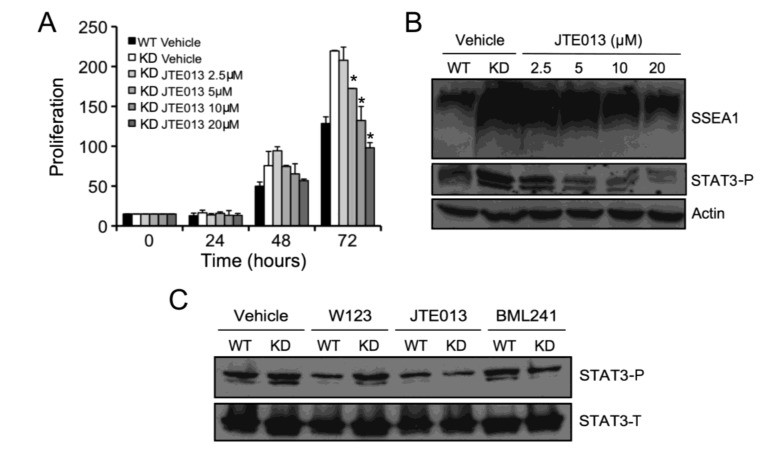
SPL silencing in mESCs activates STAT3 via S1P_2_ signaling. (**A**) SPL-KD and WT mESCs were grown to confluence, trypsinized, counted, and seeded at 75,000 cells/mL. Cells were grown in the presence of vehicle or varying concentrations from 2.5 to 20 µM of the S1P_2_ antagonist JTE013. Cells were trypsinized and harvested for cell counting in duplicate at the indicated time points; (**B**) SPL-KD and WT mESCs were treated as described in (**A**) but were harvested at 72 h incubation, and evaluated by immunoblotting of whole cell extracts to detect the pluripotency marker SSEA1, and phosphorylated STAT3. Actin was used as a loading control; (**C**) SPL-KD and WT mESCs were grown to confluence, trypsinized, counted, and seeded at 75,000 cells/mL. Cells were grown in the presence of vehicle or the S1P receptor antagonists W123 (S1P_1_), JTE013 (S1P_2_) and BML241 (S1P_3_). Cells were trypsinized, and whole cell extracts were evaluated for total and phosphorylated STAT3 proteins. * in (**A**) For KD plus JTE013 at 5, 10 and 20 µM *vs.* vehicle, *p <* 0.05.

To confirm the role of S1P_2_ in regulating mESC proliferation, we compared the proliferation rates of SPL-KD mESC cultures treated with increasing concentrations of S1P_2_ antagonist JTE013 from 2.5 to 20 µM and incubated for 72 h. JTE013 treatment resulted in a dose-dependent attenuation in proliferation rate of SPL-KD mESCs, resulting in a rate similar to that of WT mESCs (Figure 5A). Similarly, with increasing concentrations of JTE013, the high expression of pluripotency marker SSEA1 was reduced in SPL-KD cells in a dose-dependent manner to levels similar to those of WT mESCs (Figure 5B). In addition, STAT3 activation, as determined by the level of STAT3-P, was reduced in dose-dependent fashion by S1P_2_ antagonism (Figure 5B). Similarly, OCT4 expression was reduced in response to S1P_2_ antagonist treatment (Figure 5C). The S1P_1_ antagonist W123 had no effect on OCT4 expression, whereas the S1P_3_ antagonist BML241 appeared to have some effect. These findings suggest that SPL silencing activates STAT3 signaling through an S1P_2_-dependent pathway and thereby promotes mESC proliferation and pluripotency. 

### 2.6. SPL-KD Cells are Capable of Generating Tissue Stem Cells

**Figure 6:**
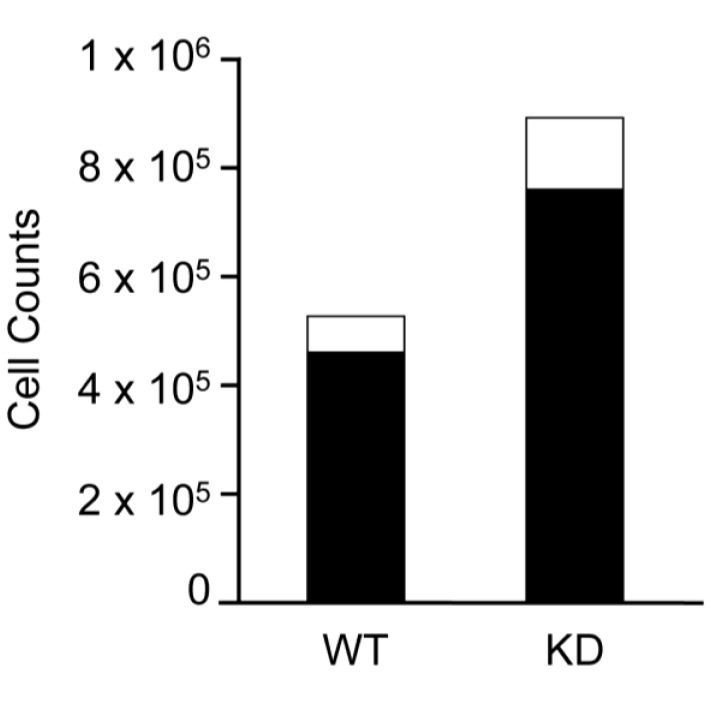
SPL-KD cells are capable of generating satellite cells. WT and SPL-KD mESC cell lines were induced to form embryoid bodies using the hanging drop method. Cells were dissociated, and satellite cells were isolated from total cells using a FACSAria cell sorter to separate cells positive or negative for the monoclonal antibody SM/C-2.6, which recognizes an antigen on the cell surface of satellite cells. Total number of viable cells counted is shown in black bars, and SM/C-2.6+ cells are shown in white bars for each line.

It became important to determine whether inhibition of SPL hinders the capacity of mESCs to give rise to tissue stem cells and thus be of utility in cell therapy schemes. Toward that end, we generated embryoid bodies from WT and KD mESC cultures using the hanging drop method. We then dissociated the embryoid bodies into single cell suspensions and quantified the percentage of skeletal muscle stem cells called satellite cells using a novel monoclonal antibody, SM/C-2.6. As shown in Figure 6, 15% of WT embryoid body cells were positive for SM/C-2.6, and a comparable 18% of KD embryoid body cells were positive for the marker. These findings suggest that SPL inhibition does not hinder the formation of murine tissue stem cells from mESCs.

### 2.7. Discussion

In this study, we explored the role of the S1P catabolic enzyme SPL in mESC biology by generating mESCs in which SPL is silenced through shRNA expression and comparing biochemical and functional endpoints with WT controls. We show that robust SPL expression is present in WT mESCs. When SPL expression is inhibited, intracellular S1P levels increase. In addition, proliferation rates and expression of the pluripotency markers OCT4 and SSEA1 increase when SPL is silenced. Loss of expression of SSEA1, and concomitant decreases in expression of pluripotency transcription factors is indicative of an overall decrease in the pluripotent potential of mESCs and a shift towards differentiation [31,32]. OCT4 is a transcription factor belonging to the POU family of transcription factors. Its expression is restricted to the developing mouse embryo, and expression decreases upon differentiation of the epiblast. Appropriate levels of OCT4 expression are critical to maintenance of pluripotency; decreases in expression lead to shifts toward a trophoectodermal cell fate, while increases in expression lead to differentiation of ESCs toward an endodermal or mesodermal cell fate [33]. We do not know the mechanism by which pluripotency marker expression is altered in SPL-KD cells, although the effects appear to be downstream of S1P_2_ activation, and effects on SSEA1 are dependent upon STAT3 activation. We did not measure the effects of SPL, S1P_2_ or STAT3 on pluripotency marker protein stability or gene message levels. Thus, we cannot comment on whether the effects are transcriptional or post-transcriptional. STAT3 motifs found in human OCT4, NANOG and SOX2 promoters are not present in the corresponding murine genes, so we do not suspect SPL mediates its effects via a direct transcriptional regulatory mechanism involving STAT3-dependent gene induction. Pluripotency marker expression levels may be an indirect consequence of effects of S1P signaling that lead to maintenance of the undifferentiated state or prevention of differentiation in mESCs. SSEA1 was more profoundly affected by SPL expression status than OCT4. SSEA1 is a large-molecular-mass glycoprotein that contains a glycosphingolipid epitope. It is possible that accumulation of sphingolipid metabolites in the absence of SPL may enhance their incorporation into complex sphingolipids such as SSEA1. Further studies will be required to establish the specific mechanism by which SPL exerts its effects on mESC pluripotency marker expression.

Increased proliferation, OCT4 and SSEA1 expression in association with suppression of SPL in our cell model suggests that the modulation of SPL expression in ESCs may provide an advantage in cell expansion protocols for tissue-specific cell therapies. In that regard, we have also observed upregulation of OCT4 expression in human ESCs in which SPL is silenced by shRNA-mediated knockdown (data not shown). Our finding of increased SSEA1 and OCT4 expression but no detectible impact on SOX2 and NANOG expression in response to SPL silencing suggests that SPL differentially regulates factors critical to stem cell homeostasis. The ultimate impact of SPL silencing on *ex vivo* expansion and differentiation into ectoderm, endoderm and mesoderm and tissues deriving from them will require further study of these mESC lines as well as corresponding hESC lines under a variety of proliferation and differentiation-promoting conditions. We suspect that S1P generated and accumulated in SPL-KD cells is exported to the extracellular environment, where it can bind to receptors on the cell surface thereby mediating the effects we have observed in mESCs. Our finding that S1P_2_ plays a role in mediating the SPL-KD cell phenotype and STAT3 activation in mESCs suggests that the effect is not mediated by the known intracellular functions of S1P. However, we cannot exclude a role for intracellular receptor-mediated signaling in this process. Considering the impact that SPL silencing has on mESC biology, it is possible that other enzymes involved in S1P biosynthesis or degradation such as sphingosine kinases, S1P phosphatases or other lipid phosphatases may play important roles in this process as well. 

STAT3 signaling is critical to mESC proliferation and maintenance of pluripotency, and downregulation of STAT3 signaling in mESCs results in differentiation and loss of expression of master pluripotency transcription factors OCT4, SOX2 and NANOG [28]. In human ESCs, S1P treatment is shown to upregulate ERK1/2 signaling [22], yet S1P/ERK signaling is not well understood in the mouse model. Furthermore, the role of S1P in STAT3 signaling, a known downstream LIF signaling mechanism, is not well understood in mESC biology. In our study, inhibition of ERK1/2 signaling through use of the MEK1 inhibitor PD98059 led to a slight increase in cell proliferation in both WT and SPL-KD mESCs, consistent with data showing that ERK signaling through a gp130-dependent mechanism is not necessary for mESC self-renewal [23]. Following inhibition of PI_3_K signaling, complete ablation of growth was observed in both control and SPL-KD mESCs, suggesting that PI_3_K signaling is absolutely critical to mESC survival, that S1P intracellular signaling does not likely occur through PI_3_K networks, and that the proliferative difference in WT and SPL-KD mESCs does not involve a PI_3_K-dependent signaling mechanism. However, inhibition of STAT3 signaling resulted in decreased proliferation in both control and SPL-KD mESCs, and STAT3 inhibition decreased levels of SSEA1, a marker of pluripotency, in SPL-KD mESCs. Importantly, we consistently observed that STAT3 activation was more pronounced in SPL-KD than WT mESCs as shown by phosphorylation on Tyr705. Taken together, these results implicate STAT3 signaling in mediating the regulation of mESC pluripotency by SPL.

We observed expression of all five S1P receptors in E14 mESCs. Others have also observed that mESCs express multiple S1P receptors [34]. However, our findings suggest that alterations of S1P metabolism can influence the relative gene expression of individual receptor subtypes, as shown by a reduction of S1P_1_ and an increase in S1P_2_ in SPL-KD compared to WT control mESCs. Cell surface expression of S1P_1_ is sensitive to the local levels of S1P in the media. However, this would not explain our finding, which instead suggests the existence of a feedback mechanism for regulating S1P receptor gene expression that is influenced by intracellular or extracellular S1P levels. Importantly, we observed that SPL silencing promoted cell proliferation, SSEA1 expression and STAT3 activation in an S1P_2_-dependent manner. This finding is similar to our observations of SPL-dependent effects on muscle stem cells [35]. In multipotent hematopoietic stem cells, S1P/ S1P_2_ signaling is thought to critical to regulation of proliferation [36]. When antagonists of S1P_1_, S1P_2_, and S1P_3_ were applied to WT and SPL-LD mESCs, we observed significant decreases in proliferation of both control and SPL-KD mESCs during S1P_2_ antagonism, compared to little difference in growth in the presence of S1P_1_ and S1P_3_ receptor inhibitors, *versus* controls. Furthermore, western blots with cell extracts grown in the presence of S1P_2_ antagonist showed decreased expression of SSEA1 in conjunction with decreased phospho-STAT3 (Tyr705). Altogether, our findings demonstrate that SPL is expressed in ESCs, and its activity controls STAT3-dependent effects on cell proliferation and pluripotency by promoting S1P signaling through S1P_2_. These findings demonstrate an important role for SPL in ESC homeostasis and suggest that SPL inhibition could potentially facilitate *ex vivo* ESC expansion for therapeutic purposes. Although currently available SPL inhibitors are not efficacious *in vitro*, the crystal structure of SPL is now available, and additional inhibitors are likely to be developed in the near future. 

## 3. Experimental Section

### 3.1. Embryonic Stem Cell Culture

E14 mESCs were grown at 37 °C with 5% CO_2_ on gelatin-coated plates in Glasgow’s Minimum Essential Media (Sigma Aldrich) supplemented with 10% ES screened Fetal Bovine Serum (Hyclone Thermo Scientific) and 10^4^ units/mL ESGRO LIF (Millipore, Temecula, CA, USA). Sodium Pyruvate (1 mM), Non-Essential Amino Acids (0.1 mM), Penicillin (100 units/mL), Streptomycin (100 µg/mL) and L-Glutamine (2 mM) (UCSF Cell Culture Facility) and 2-mercaptoethanol (4 µM) (Sigma Aldrich) were added to the media. WT and SPL-KD mES cells were grown to confluence, trypsinized, counted, and seeded at varying densities from 50,000 cells/mL to 150,000 cells/mL. Differences in proliferation rates between the two cell lines were observed under each of these conditions. Therefore, for consistency, all additional studies were conducted using a density of 75,000 cells/mL.

### 3.2. SPL Knockdown

The lentiviral vector pLKO.1 (Addgene plasmid 10878; Addgene, Cambridge, MA, USA) was used to clone shRNAs targeting the human SPL gene *Sgpl1* according to pLKO.1 protocol (The virions were produced by transfection of HEK293T ). The virions were produced by transfection of HEK293T cells using the recombinant pLKO.1 along with packaging plasmid psPAX2 (Addgene plasmid 12260) and the envelope-encoding plasmid pMD2.G (Addgene plasmid 12259). Forty-eight hours post-transfection, the spent media containing the lentiviral particles was collected. This was used to infect Sgpl^+/−^ cells in the presence of 8 μg/mL polybrene. Stable clones were selected with puromycin (2 μg/mL). The surviving colonies were screened for mouse SPL expression levels using western blotting. Similar studies were performed to knockdown SPL in Sgpl1^+/+^ mESCs. However, complete silencing was not achieved, and no phenotype was found to be associated with partial knockdown. Therefore, all experiments performed in this study utilize the SPL-KD cells developed using the Sgpl1^+/−^ starting mESC line. Several independent knockdown experiments were performed to obtain multiple clones of vector control and SPL-KD paired lines. All results have been verified in more than one pair of mESC control and knockdown lines. 

### 3.3. SPL Assay

SPL enzymatic assay was performed as described previously [37]. Briefly, cells were grown in gelatin coated 6-well plate and allowed to reach 80% confluency. Then, cell lysate from WT and SPL-KD mESCs (equivalent to 25 µg protein) was incubated with NBD-labeled S1P [Omega(7-nitro-2-1,3-benzoxadiazol-4-yl)-D-*erythro*-sphingosine-1-phosphate]; Avanti Polar Lipids , Cat# 810207X) for 45 min at 37 °C. After completion of reaction, NBD-labeled sphingosine was added as an internal standard, followed by potassium hydroxide chloroform extraction. Organic phase was collected and the NBD-labeled product of the SPL reaction (hexadecenal) was detected by HPLC using C18 column.

### 3.4. Mass Spectrometry

S1P levels from WT and SPL-KD mESCs were measured by mass spectrometry as described previously [35]. Briefly, cells were grown in gelatin coated 6-well plate and allowed to reach 80% confluency. For S1P measurement, WT and SPL-KD mESCs were pooled of each type from 3 wells of 6-well plate as S1P was extracted using chloroform:methanol. Lipids were separated on a 2.1 × 50 mm Kinetex C18 column (Phenomenex, Torrance, CA, USA) at a flow rate of 0.25 mL/min. The gradient used was from 45% to 99% methanol containing 1% acetic acid and 5 mM ammonium acetate. The data were acquired in positive mode on a Micromass Quattro LCZ (Waters Corp.) mass spectrometer. Lipids were identified based on their specific precursor and product ion pair and quantified using multiple reaction monitoring. Commercial 17C-S1P was used as an internal standard.

### 3.5. Western Blotting

Immunoblotting was performed as described [35]. Antibodies to SSEA1 (MC450), SOX2 (L73134), OCT4 (C30A3), STAT3 (124H6), phospho-STAT3 (Tyr705) (3E2), and HRP conjugated anti-rabbit antibodies were from Cell Signaling Technologies (Beverly, MA, USA). NANOG (AB5731) antibody was from Millipore. Antibody to actin was from Sigma. Immunoblotting for SPL was performed using a rabbit polyclonal antibody generated against the C-terminal peptide of murine SPL of the sequence: C-VTQGNQMNGSPKPR. Murine SPL antibody was used at 1:1,000 dilution. 

### 3.6. Cell Proliferation Assays

WT and SPL-KD mES cells were grown to confluence, trypsinized, counted, and seeded at 75,000 cells/mL. Cells were grown in the presence of the MEK1 inhibitor PD98059 (Selleck Chemicals, Houston, TX, USA), PI_3_K inhibitor LY294002 (CalBiochem, Billerica, MA, USA), STAT3 inhibitor Stattic (Santa Cruz Biotech, Santa Cruz, CA, USA), S1P_1_ receptor antagonist W123, S1P_2_ receptor antagonist JTE013, and S1P_3_ receptor antagonist BML241 (Cayman Chemicals, Ann Arbor, MI, USA). Fresh media and inhibitors were added at 24-h time points. For live cell counts, cells were trypsinized and counted in duplicate at 24-h time points by hemocytometer and with a Beckman Coulter cell counter. For cytotoxicity, media was removed, centrifuged at 2000 rpm and cells were resuspended. Adherent cells were trypsinized, centrifuged and added to the floating cell suspension. Dead cells were quantified with 0.04% Trypan Blue using a hemocytometer. 

### 3.7. RT-PCR

Total RNA was isolated from cells using Trizol reagent (Invitrogen, Carlsbad, CA, USA) according to the manufacturer’s instructions. RT-PCR was performed using the one-step RT-PCR kit (Qiagen, Valencia, CA, USA). Following primers were used for mouse: S1P_1_ forward 5’-ACCTAGCCCTCTCGGACCTATT-3', S1P_1_ reverse 5’- CCCAGACAACAG CAGGTTAGC-3', S1P_2_ forward 5'-GCCATCGCCATCGAGAGA-3’, S1P_2_ reverse 5’-TGTCACTGCCGTAGAGCTTGA-3’, S1P_3_ forward 5’-GCCAGTCTTGGGAAATGACACT-3', S1P_3_ reverse 5’-TGCCAGTTTCCCCACGTAA-3', S1P_4_ forward 5’-CTGCCCGCCGCAAGT-3’, S1P_4_ reverse 5’-CACAAAGGCCACCAAGATCA-3', S1P_5_ forward 5’-TGGCTGTGTGTGCCTTCATT-3’, S1P_5_ reverse 5’-GCGGACCAGCACCAAGAG-3’actin forward 5’-AGAAAATCTGGCACCACACC-3’ and actin reverse 5’-AGAGGCGTACAGGGATAGCA-3’. 

### 3.8. Isolation of Satellite Cells from mESC Embryoid Bodies

WT and KD mESCs were induced to form embryoid bodies using the hanging drop culture method as described previously [38]. Embryoid bodies were incubated in enzyme-free Hank’s-based Cell Dissociation Buffer (Invitrogen) for 30 min at 37 °C, then gently dissociated into a single cell suspension. Cells were subjected to centrifugation, washed twice with PBS, and labeled with biotinylated-SM/C-2.6 antibody (0.2 mg/mL, diluted to < 1:200) for 15 min at room temperature [39]. Cells were then stained with Streptavidin-ALEX 647 for 15 min at room temperature and incubated in 1 µg/mL propidium iodide solution for 5 min. Cells were sorted and counted on a Becton Dickinson FACSAria I cell sorter.

### 3.9. Statistical Analysis

Statistical analysis was performed using Student’s *t* test. *P* value ≤ 0.05 was considered significant.

## 4. Conclusions

Our findings are suggestive of an S1P-S1P_2_ receptor-STAT3 signaling mechanism in mESCs that serves to regulate both growth and pluripotency networks. Western blots for mESCs grown in the presence of S1P_1_ and S1P_3_ receptor inhibitors showed no difference in SSEA1 or phospho-STAT3 expression, suggesting that neither S1P_1_ nor S1P_3_ receptors serve as major regulators of signaling networks that regulate mESC pluripotency or growth. Furthermore, proliferation studies with WT and SPL-KD mESCs grown in the presence of S1P_2_ antagonist JTE013 at a variety of concentrations show a dose-response growth inhibition effect without any significant increase in necrotic cell death, indicating that this effect is not due to non-specific cytotoxic effects. Increasing the dosage of S1P_2_ antagonist (5–20 µM) led to a dose-dependent decrease in phospho-STAT3 levels, evidence that S1P_2_ receptor signaling likely functions through a STAT3-dependent mechanism. Furthermore, SSEA1 levels are decreased in a similar dosage-dependent manner in the presence of S1P_2_ receptor inhibition, indicating that expression of mESC pluripotency markers are dependent on an S1P_2_-STAT3 dependent signaling pathway. Elucidating the downstream network of signaling from the S1P_2_ receptor to STAT3 signaling is among one of the next steps of research necessary to understand how SPL regulates proliferation and pluripotency of mESCs.
